# The influence of college students’ “Wan Geng” motivation on emotion regulation self-efficacy

**DOI:** 10.3389/fpsyg.2026.1802652

**Published:** 2026-07-17

**Authors:** Ziyue He, Yawen Zhao, Yiming Shan, Fuyu Wan, Tour Liu

**Affiliations:** 1 Department of Journalism and Communication, Tianjin Normal University, Tianjin, China; 2 Faculty of Psychology, Tianjin Normal University, Tianjin, China; 3 Key Research Base of Humanities and Social Sciences of the Ministry of Education, Academy of Psychology and Behavior, Tianjin Normal University, Tianjin, China; 4 Tianjin Key Laboratory of Student Mental Health and Intelligence Assessment, Tianjin Normal University, Tianjin, China

**Keywords:** behavioral motivation, college students’ “Wan Geng” behavior, emotion regulation, emotional regulation self-efficacy, psychological comfort

## Abstract

“Wan Geng” (玩梗) refers to the playful creation, remixing, and sharing of internet memes. While a global online behavior, it carries distinctive linguistic features in the Chinese cultural context and has become an important way for young people to express emotions and shape identity. Drawing on Uses and Gratifications Theory, Emotion Regulation Theory, and Self-Efficacy Theory, this study examined how different “Wan Geng” motivations relate to emotion regulation strategies and emotion regulation self-efficacy among university students. Using convenience sampling, an online survey was conducted with 1,084 valid participants, who completed the College Students’ “Wan Geng” Motivation Scale, the Emotion Regulation Questionnaire, and the Regulatory Emotional Self-Efficacy Scale. Data were analyzed using SPSS and Mplus. Three findings emerged: (1) entertainment and group integration motivations were positively associated with emotion regulation self-efficacy; (2) “Wan Geng” motivations were linked to emotion regulation strategies; (3) entertainment, self-identity, and group integration motivations were indirectly associated with emotion regulation self-efficacy via emotion regulation strategies, while emotion regulation and entertainment motivations showed direct associations. These findings suggest that “Wan Geng” may constructively associated with university students’ emotion regulation, offering a fresh perspective on how culturally embedded online behavior relates to psychological well-being.

## 1 Introduction

“Wan Geng” (玩梗), literally “playing with memes” refers to the creative, context-sensitive use and remixing of internet memes for communication, humor, and emotional expression. As a global participatory digital practice, “Wan Geng” involves the circulation and creative transformation of culturally shared units (梗, gěng). However, in the Chinese context is distinguished by its deep roots in local linguistic features, such as homophonic puns, dialect-based humor, and “inside jokes” tied to social events, making it both a universal online behavior and a culturally specific practice. Unlike passive browsing or simple sharing, “Wan Geng” requires deliberate reinterpretation and strategic use in social interactions. In this sense, it serves not only as a vehicle for humor, but also as a means of emotional expression and group bonding. In China, university students are among the primary users and disseminators of this practice. According to a China Youth Daily survey (2024), 95.67% of surveyed college students reported engaging in “Wan Geng,” among them, 65.62% felt it makes communication more efficient and enjoyable and is thereby seen as fostering social interaction, and 62.03% regarded it as a means to relieve stress, relax, and express emotions promptly. At the same time, the role of “Geng” is changing: online interactions have shifted from attracting attention to mobilizing emotions, and even to guiding emotions strategically—a trend illustrated by Oxford’s 2025 Word of the Year, “rage bait” (Shin et al., 2025). Although “Wan Geng” can occasionally expose individuals to emotional contagion and impression management pressures, its prevalent use for mood relief and social connection underscores the importance of studying the emotion regulation processes associated with this digital behavior.

The popularity of “Wan Geng” can be explained through Uses and Gratifications (U&G) theory, which views individuals as active audiences who select and use media to meet specific psychological and social needs (Katz et al., 1974; Whiting and Williams, 2013). For university students, “Wan Geng” reflects a combination of psychological demands. Building on U&G theory, this study identifies four core motivations behind this behavior: emotion regulation (relieving stress and managing mood), entertainment (seeking fun and relaxation), self-identity (expressing personal values and shaping self-image), and group integration (connecting with peers and building group identity). Through actively selecting, creating, and sharing memes, students fulfill these needs in ways that may be associated with temporary psychological relief and stronger social bonds. However, while U&G theory explains why students engage in “Wan Geng,” it does not fully account for how these motivations are associated with emotion regulation. In particular, it does not address how the gratifications gained from “Wan Geng” may relate to individuals’ beliefs in their ability to manage emotions.

This study draws on Bandura’s Self-Efficacy Theory. It focuses on regulatory emotional self-efficacy (RESE), which refers to one’s belief in the ability to manage emotional states (Bandura et al., 2003). It also applies Emotion Regulation Theory (Gross, 2015) to position emotion regulation strategies as the mediating mechanism. Because two of the four motivations involve identity and belonging, this study further draws on Social Identity Theory to interpret the self-identity and group integration pathways. Together, these frameworks form the basis of an integrated conceptual model.

According to Self-Efficacy Theory, successful experiences are the most powerful source of efficacy beliefs. Stronger “Wan Geng” motivations may be associated with more frequent engagement in this practice, providing repeated opportunities to manage emotions in a socially embedded digital context. Emotion Regulation Theory identifies two widely used strategies: cognitive reappraisal and expressive suppression. Research indicates that individuals who use these strategies more effectively tend to reinterpret emotional stimuli and adjust their emotional states more readily (Wan et al., 2024; Alessandri et al., 2023). In “Wan Geng” interactions, students face challenges such as emotional contagion and impression management, but also find opportunities for group identification and emotional release. When they use reappraisal to humorously reframe stressful content, or suppression to align with group norms, they may experience a sense of mastery. Self-Efficacy Theory posits that such mastery experiences strengthen efficacy beliefs; accordingly, these positive experiences may be associated with stronger efficacy beliefs, especially in online environments that require stress management or social connection (Verduyn et al., 2020). In addition, students tend to use cognitive reappraisal and expressive suppression in consistent ways (McRae and Gross, 2020), and more favorable attitudes toward these strategies are linked to more frequent use (Donker et al., 2020).

Thus, this study proposes the following pathway: “Wan Geng” motivation may be associated with greater use of emotion regulation strategies, and more effective use of these strategies may, in turn, be associated with higher regulatory emotional self-efficacy. This process-oriented pathway has not been empirically tested in prior research, which has mostly focused on meme diffusion patterns or general gratifications from meme consumption. Based on this theoretical integration, the present study tests a mediation model with emotion regulation strategies as the central mediator. The model examines the pathway from “Wan Geng” motivation to regulatory emotional self-efficacy and clarifies how this behavior may relate to university students’ psychological well-being. Two hypotheses are proposed accordingly.

*H*1: University students’ “Wan Geng” motivation is positively associated with their regulatory emotional self-efficacy.

*H*2: Emotion regulation strategies mediate the relationship between “Wan Geng” motivation and emotion regulation self-efficacy.

## 2 Methods

### 2.1 Participants

This study focused on university students as the research participants. Data were collected through an online survey using convenience sampling, administered from March 22 to April 10, 2025. The survey was hosted and distributed through Wenjuanxing (问卷星), one of the most widely used online survey platforms in China. Participants accessed and completed the questionnaire via a shared link or QR code. The questionnaire combined a self-developed “Wan Geng” motivation scale with two established psychometric instruments, Emotion Regulation Scale and Regulatory Emotional Self-Efficacy Scale, and was administered in Chinese. A total of 1,478 responses were initially received. After screening for completeness and removing outliers, 1,084 valid questionnaires were retained for analysis, including the 265 participants used to develop and validate the self-developed “Wan Geng” motivation scale. The final sample comprised 762 female (70.30%) and 322 male (29.70%) participants, covering all 14 disciplinary categories defined by the Ministry of Education of China.,providing diversity across gender and academic discipline. It should be noted that the use of convenience sampling through an online platform may have introduced certain sampling biases, as the sample may overrepresent students who are more active online or more willing to participate in voluntary surveys. The study was reviewed and approved by the Research Ethics Board of Tianjin Normal University (Approval No. 2024030431). All participants provided informed consent prior to participation. Data analysis was performed using SPSS 26.0 for preliminary data processing, the PROCESS macro for SPSS to examine mediation effects, and Mplus 8.3 for mediation model testing.

### 2.2 Instruments

#### 2.2.1 Motivation scale for college students’ “Wan Geng” behavior

A self-developed scale was used to measure participants’ motivations for engaging in “Wan Geng.” Grounded in Uses and Gratifications Theory (Palmgreen, 1984; Ruggiero, 2000), the scale was adapted from the Online Reading Motivation Questionnaire (Mondi et al., 2008) and the We Chat “Liking” Behavior Motivation Scale (Rong and Ke, 2015). The validation process included item analysis, exploratory factor analysis, and confirmatory factor analysis, conducted using SPSS 30.0 and Mplus 8.3. The final 14-item scale consists of four dimensions: emotion regulation motivation, entertainment motivation, self-identity motivation, and group integration motivation. All retained items showed loadings above the recommended threshold of 0.5. Responses were recorded on a 5-point Likert scale (1 = Strongly Disagree, 5 = Strongly Agree), with higher scores indicating stronger motivation in each dimension. The scale demonstrated excellent internal consistency (Cronbach’s *α* = 0.945) and structural validity (KMO = 0.949; Bartlett’s test of sphericity: χ^2^ = 1023.56, *p* < 0.001), with test–retest reliability of 0.927** (*p* < 0.01). Model fit indices (CFI = 0.948, TLI = 0.933) further confirmed the scale’s appropriateness for large-scale assessment.

#### 2.2.2 Emotion regulation scale

The use of emotion regulation strategies was assessed using the self-report scale developed by Gross and John (2003),which was later adapted into a Chinese version by Wang et al. (2007). The scale consists of 10 items, rated on a 7-point Likert scale, with higher scores indicating more frequent use of emotion regulation strategies. In this study, the scale demonstrated good internal consistency, with a Cronbach’s *α* coefficient of 0.836.

#### 2.2.3 Regulatory emotional self-efficacy scale

Emotional self-efficacy was measured using a scale developed by Caprara et al. (2008) and adapted into Chinese by Wang et al. (2013). The scale employs a second-order five-factor structure, which includes the dimensions of “self-efficacy in expressing positive emotions” and “self-efficacy in managing negative emotions.” Responses were recorded on a 5-point Likert scale (1 = Strongly Disagree, 5 = Strongly Agree), with higher scores indicating greater emotional self-efficacy. In the current study, the scale showed excellent internal consistency, with a Cronbach’s *α* coefficient of 0.917.

This study constructed a parallel mediation model with the four dimensions of “Wan Geng” motivation (emotion regulation, entertainment, self-identity, and group integration) as independent variables, the two emotion regulation strategies (cognitive reappraisal and expressive suppression) as mediators, and regulatory emotional self-efficacy as the dependent variable. Mediation effects were tested using the PROCESS macro for SPSS with 5,000 bootstrap samples, and indirect effects were considered significant when the 95% confidence interval did not include zero. Direct and indirect paths were estimated simultaneously, with no additional covariates included in the main analysis.

## 3 Results

### 3.1 Common method bias test

Harman’s single-factor test was conducted to assess potential common method variance in the data. The results indicated that the first factor accounted for 18.65% of the total variance, which is well below the conventional threshold of 40%. No single dominant factor or unusually high explained variance was observed. These findings suggest that common method bias is not a substantial concern in the present dataset, thereby supporting the credibility of the observed relationships among variables.

### 3.2 Relationship between college students’ “Wan Geng” motivation and regulatory emotional self-efficacy

#### 3.2.1 Correlation analysis results between variables

Table 1 reports the correlation results. Cognitive reappraisal was significantly and positively correlated with emotion regulation motivation, self-identity motivation, and group integration motivation, whereas expressive suppression was significantly and positively correlated with group integration motivation. Emotion regulation strategies were also positively correlated with emotion regulation self-efficacy. The pattern was different for “Wan Geng” motivation. The overall composite score showed no significant correlation with emotion regulation self-efficacy. At the sub-dimension level, however, two sub-dimensions, entertainment motivation and group integration motivation, each showed a significant positive correlation with self-efficacy.

**Table 1:** Correlation analysis among research variables.

Variables	1	2	3	4	5	6	7	8	9	10
1. ERM	-	-	-	-	-	-	-	-	-	-
2. EM	0.615^**^	-	-	-	-	-	-	-	-	-
3. SIM	0.503^**^	0.523^**^	-	-	-	-	-	-	-	-
4. GIM	0.401^**^	0.412^**^	0.610^**^	-	-	-	-	-	-	-
5. CR	0.055	0.102^**^	0.059*	0.146^**^	-	-	-	-	-	-
6. ES	0.068^*^	0.048	0.023	0.107^**^	0.401^**^	-	-	-	-	-
7. SE-EPE	−0.026	0.125^**^	0.047	0.092^**^	0.288^**^	−0.190^**^	-	-	-	-
8. SE-MNE	−0.02	0.032	0.048	0.034	0.264^**^	−0.008	0.396^**^	-	-	-
9.”Wan Geng”M	0.767^**^	0.770^**^	0.853^**^	0.786^**^	0.114^**^	0.077^*^	0.074^*^	0.032	-	-
10. ERSE	−0.026	0.076^*^	0.056	0.064^*^	0.320^**^	−0.084^**^	0.715^**^	0.925^**^	0.055	-

*p < 0.05, **p < 0.01, two-tailed test.

A likely explanation lies in the multidimensional structure of “Wan Geng” motivation. The composite score is an average of four sub-dimensions, and these sub-dimensions are associated with self-efficacy in different ways. Two sub-dimensions are positively correlated with self-efficacy, while the others are not. When all four are combined into a single score, the meaningful associations are diluted by the non-significant ones, so the composite does not reflect the patterns visible at the sub-dimension level. It suggests that “Wan Geng” motivation is better examined through its individual sub-dimensions than through its aggregate score. Accordingly, the subsequent mediation models were specified at the sub-dimension level, so that the distinct associations of each facet could be retained.

#### 3.2.2 The mediating role of emotion regulation strategies in the relationship between college students’ “Wan Geng” motivation and regulatory emotional self-efficacy

**Figure 1:**
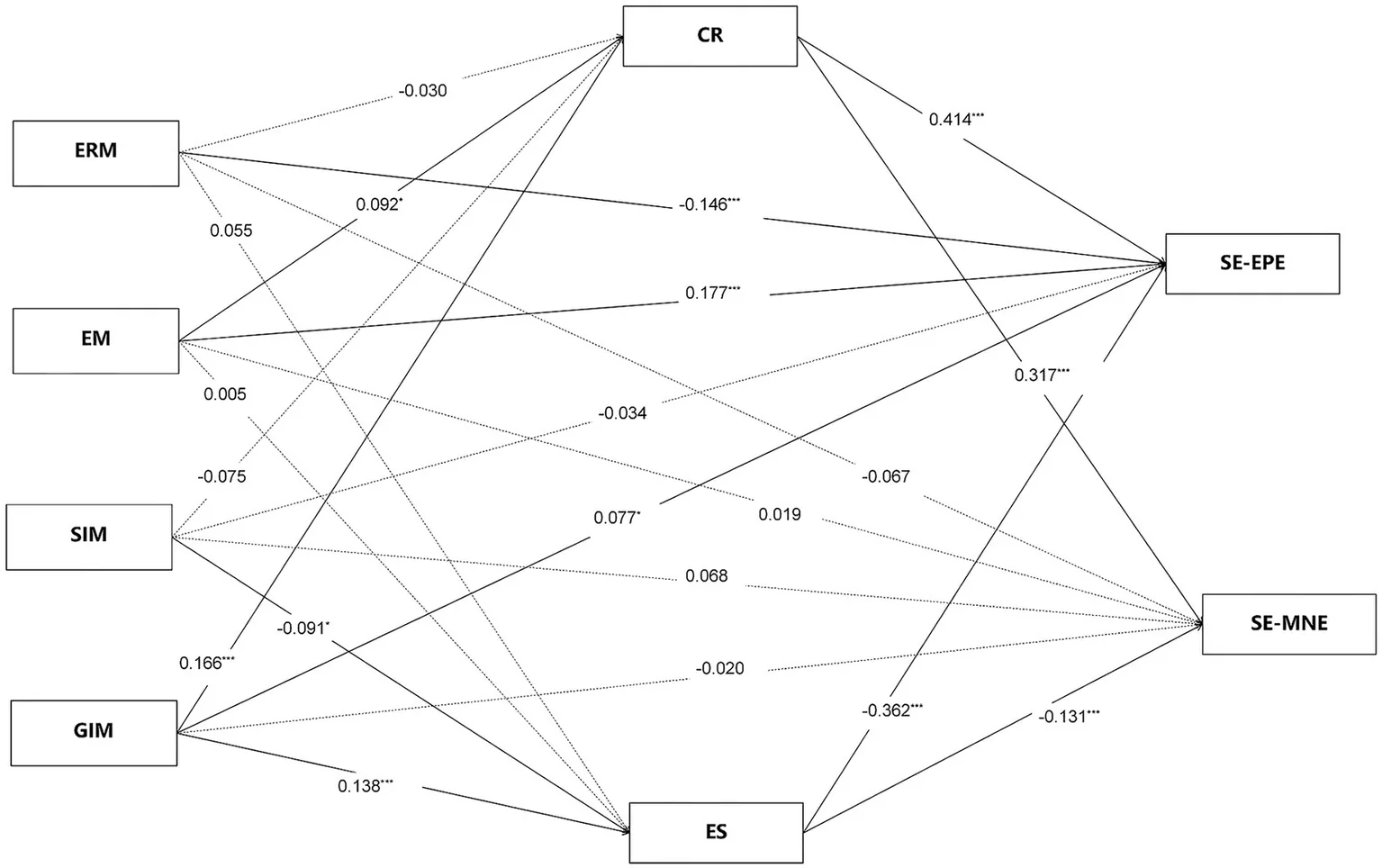
The mediating role of emotion regulation strategies between college students’ “Wan Geng” motivation and regulatory emotional self-efficacy dashed lines indicate non-significant path coefficients. Solid lines indicate significant path coefficients. *p < 0.05, ***p < 0.001.

Building on the correlation findings above, this study specified a sub-dimensional model. The four “Wan Geng” motivation dimensions (emotion regulation, entertainment, self-identity, and group integration) served as independent variables, cognitive reappraisal and expressive suppression as parallel mediators, and the two facets of regulatory emotional self-efficacy (self-efficacy in expressing positive emotions and self-efficacy in managing negative emotions) as the dependent variables. The results are presented in Figure 1. Because all tested models were saturated, model fit indices are not reported separately.

For the direct path,emotion regulation motivationwas directly and negatively associated with self-efficacy in expressing positive emotions (*β* = −0.146, *p* < 0.001), whereas entertainment motivation was directly and positively associated with this outcome (*β* = 0.177, *p* < 0.001) In contrast, self-identity motivation was directly and positively associated with self-efficacy in managing negative emotions (*β* = 0.077, *p* < 0.05). Additionally, cognitive reappraisal was positively associated with both forms of self-efficacy (*β* = 0.414 and 0.317, both *p* < 0.001), whereas expressive suppression was negatively associated with both (*β* = −0.362, *p* < 0.001; *β* = −0.131, *p* < 0.001). In addition, group integration motivation was positively associated with both strategies (*β* = 0.166 and 0.138, both *p* < 0.001), entertainment motivation with cognitive reappraisal only (*β* = 0.092, *p* < 0.05), and self-identity motivation with expressive suppression only (*β* = −0.091, *p* < 0.05).

Using the Bootstrap method with 5,000 resamples, mediation analyses were conducted. As presented in Table 2, the indirect associations revealed two contrasting patterns. All four pathways mediated by cognitive reappraisal were positive: both entertainment motivation and group integration motivation indirectly predicted higher self-efficacy in expressing positive emotions and in managing negative emotions through cognitive reappraisal, with the strongest effect observed for group integration motivation → cognitive reappraisal → expressing positive emotions (*B* = 0.069, 95% CI [0.034, 0.105], effect proportion = 71.9%). In contrast, the four pathways via expressive suppression showed mixed directions: group integration motivation was negatively associated with both forms of self-efficacy through expressive suppression, whereas self-identity motivation was positively associated with both. Notably, three pathways exhibited inconsistent mediation (MacKinnon et al., 2000), for which the interpretation of the effect proportion does not apply, and discussed in detail later (marked with “--ᵃ” in Table 2).

**Table 2:** The mediating role of emotion regulation strategies between regulatory emotional self-efficacy and motivation of college students’ “Wan Geng” behavior.

Approach	*β*	*SE*	Bootstrap 95% CI		EF
			LLCI	ULCI	
ER-M → CR → SE-EPE	−0.012	0.018	−0.046	0.024	--
EM → CR → SE-EPE	0.038^*^	0.018	0.004	0.073	17.8%
SI-M → CR → SE-EPE	−0.031	0.019	−0.070	0.006	--
GI-M → CR → SE-EPE	0.069^***^	0.018	0.034	0.105	71.9%
ER-M → CR → SE-MNE	−0.010	0.014	−0.036	0.018	--
EM → CR → SE-MNE	0.029^*^	0.014	0.003	0.060	61.0%
SI-M → CR → SE-MNE	−0.024	0.015	−0.055	0.004	--
GI-M → CR → SE-MNE	0.053^***^	0.015	0.027	0.085	--^a^
ER-M → ES → SE-EPE	−0.020	0.015	−0.050	0.008	--
EM → ES → SE-EPE	−0.002	0.015	−0.032	0.028	--
SI-M → ES → SE-EPE	0.033^*^	0.016	0.001	0.067	--^a^
GI-M → ES → SE-EPE	−0.050^***^	0.015	−0.082	−0.022	52.4%
ER-M → ES → SE-MNE	−0.007	0.006	−0.022	0.002	--
EM → ES → SE-MNE	−0.001	0.006	−0.012	0.011	--
SI-M → ES → SE-MNE	0.012	0.007	0.001	0.029	21.3%
GI-M → ERS → SE-MNE	−0.018^*^	0.008	−0.037	−0.006	--^a^

SE stands for Standard Error. β is Standardized Coefficient. *p < 0.05, **p < 0.01, ***p < 0.001. 95% confidence intervals were calculated using the Bootstrap method with 5,000 samples. “--ᵃ” indicates a suppression effect, for which EF is not reported using the standard formula.

## 4 Discussion

This study aimed to clarify how “Wan Geng” motivation is associated with regulatory emotional self-efficacy and the role of emotion regulation strategies in this association. The findings indicate that certain motivations underlying “Wan Geng” behavior were associated with regulatory emotional self-efficacy, while other motivations showed an indirect association with self-efficacy in managing negative emotions via emotion regulation strategies. This pattern may reflect a mediation structure in which “Wan Geng” motivation, emotion regulation strategies, and regulatory emotional self-efficacy are interrelated among college students, and it suggests that “Wan Geng” motivation may be meaningfully associated with emotion regulation in this population. Theoretically, this study offers a new measurement tool and a fresh perspective on the motivations behind “Wan Geng” and emotion regulation in online social contexts. Practically, the findings link “Wan Geng” to college students’ emotional functioning and offer implications for mental health interventions.

### 4.1 The role of college students’ “Wan Geng” motivation on regulatory emotional self-efficacy

The findings indicate that both entertainment motivation and group integration motivation were positively associated with regulatory emotional self-efficacy. Furthermore, two motivations were directly linked to self-efficacy in expressing positive emotions, providing support for Hypothesis H1. Consistent with prior theories (Bandura, 1977; Bandura et al., 2003; Hasking et al., 2018), self-efficacy can be understood as an individual’s perception of their capacity for emotion regulation, which tends to co-occur with the behaviors and emotional responses they show when facing stressors. The humor and conciseness of “Wan Geng” have been regarded as channels through which college students seek and reinforce emotional support (Kashitsyna, 2024). Survey results also showed that most students viewed “Wan Geng” as a way to temporarily distance themselves from stressors and experience emotion regulation through immediate positive feedback in online communication. Taken together, “Wan Geng” may reflect not only a form of entertainment, but also a psychosocial behavior linked to college students’ regulatory emotional self-efficacy, particularly self-efficacy in expressing positive emotions. Notably, the findings indicate a negative correlation between emotion regulation motivation and self-efficacy in expressing positive emotions, which is inconsistent with certain previous research. Currently, negative emotions among college students often stem from multifaceted and interconnected sources, such as academic pressure, social anxiety, and future uncertainty, which tend to compound one another. According to Self-Determination Theory (Deci and Ryan, 1985; Caprara and Steca, 2005; Donker et al., 2020), intrinsic motivation allows individuals to draw on internal resources and to cope proactively, even under challenging conditions. In contrast, extrinsic motivation may leave emotional states and regulatory strategies more dependent on external feedback, which may be linked to lower self-efficacy in emotion regulation (Benfer et al., 2018). Thus, while engaging in “Wan Geng” can fulfill immediate emotional needs, such as stress relief and entertainment, and provide psychological gratification, individuals with lower emotion regulation self-efficacy may still struggle to sustain long-term emotional satisfaction or effectively recover from negative emotional states (Benfer et al., 2018; Bassi et al., 2018). Long-term reliance may also be associated with poorer psychological functioning and lower emotion regulation capacity among some students (Chen, 2025; Su et al., 2025).

### 4.2 The mediating role of emotion regulation strategies between college students’ “Wan Geng” motivation and regulatory emotional self-efficacy

The study suggests that “Wan Geng” motivation was indirectly associated with emotion regulation self-efficacy via emotion regulation strategies, aligning with Hypothesis H2. Relevant theories suggest that individuals who believe in their ability to regulate emotions are more likely to adopt effective strategies and avoid emotional distress (Sheppes and Levin, 2013). The conclusion resonates with Uses and Gratifications Theory, where college students use “Wan Geng” as an adaptive strategy to alleviate real-world stress, employing entertainment to divert attention or reframe cognition, which may help compensate for unmet emotional needs in a relatively disadvantaged societal position (Kasirye, 2022).

#### 4.2.1 The mediating role of cognitive reappraisal in the relationship between college students’ “Wan Geng” motivation and regulatory emotional self-efficacy

Cognitive reappraisal is an antecedent-focused emotion regulation strategy that modifies the interpretation of an emotional event with little cognitive cost (Gross, 2002). The findings indicate that cognitive reappraisal served as a partial mediator between college students’ “Wan Geng” motivation and regulatory emotional self-efficacy. Specifically, entertainment motivation, through cognitive reappraisal, was positively associated with both self-efficacy in expressing positive emotions and self-efficacy in managing negative emotions. This suggests that when college students engage in “Wan Geng” for entertainment purposes, the pleasure and ease experienced while browsing or sharing meme content may be associated with reframing negative experiences in a more positive light (Goldin et al., 2008; Berking and Wupperman, 2012), reflecting an unintentional form of cognitive reappraisal. This form of cognitive adjustment, accompanied by positive affect, may reflect a process that co-occurs with higher emotion regulation self-efficacy (Gross and John, 2003).

Furthermore, the pathway pattern observed for group integration motivation warrants attention. Through cognitive reappraisal, group integration motivation was positively associated with both self-efficacy in expressing positive emotions and self-efficacy in managing negative emotions. However, group integration motivation itself showed a direct negative association with self-efficacy in managing negative emotions. From the perspective of Self-Determination Theory (Deci and Ryan, 1985), group integration motivation may reflect both intrinsic and extrinsic features: students may seek the inner satisfaction of group belonging, while also valuing external recognition such as peer feedback. On one hand, interactive “Wan Geng” behaviors (e.g., liking, commenting, sharing) generate shared emotional experiences,which were linked to the easing of negative emotions triggered by external stressors (Kammeyer-Mueller et al., 2009; Korgaonkar and Wolin, 1999). On the other hand, the situational interpretation and meaning-making that take place in group interactions may provide repeated opportunities to practice cognitive reappraisal. The direct negative association, in contrast, suggests that overreliance on group feedback or peer recognition for emotion regulation may be associated with a weaker sense of autonomy in regulating one’s own emotions. When students become accustomed to easing negative emotions through group resonance, their confidence in handling such emotions alone may be lower in the absence of group support. This direct negative association offset part of the positive indirect association, resulting in a suppression effect.

#### 4.2.2 The mediating role of expressive suppression in the relationship between college students’ “Wan Geng” motivation and regulatory emotional self-efficacy

Expressive suppression is a form of response modulation, referring to the activation of self-control processes to inhibit emotional behavior (Wang and Guo, 2003). The findings indicate that expressive suppression played a complex mediating role between college students’ “Wan Geng” motivation and regulatory emotional self-efficacy. On one hand, self-identity motivation was positively and indirectly associated with both self-efficacy in expressing positive emotions and self-efficacy in managing negative emotions through expressive suppression. However, self-identity motivation showed a direct negative association with self-efficacy in expressing positive emotions, resulting in a suppression effect. This pattern may reflect that, when students engage in “Wan Geng” out of self-identity motivation, “Wan Geng” platforms provide them with a low-threshold channel for emotional connection, allowing them to experience a sense of identity resonance and social satisfaction within interest-based communities (Whiting and Williams, 2013). In this context, expressive suppression may serve as a temporary emotion management strategy—for example, deliberately delaying or adjusting immediate emotional responses in order to maintain one’s image within the group, which may be associated with a stronger sense of emotional control in specific situations.

On the other hand, group integration motivation was positively associated with expressive suppression, while expressive suppression was negatively associated with both forms of regulatory emotional self-efficacy, yielding a negative indirect association. In parallel, group integration motivation also showed a direct negative association with self-efficacy in managing negative emotions, again resulting in a suppression effect. According to Gross’s theory of emotion regulation, expressive suppression consumes cognitive resources and may interfere with the performance of other cognitive tasks. Although it can reduce the outward expression of emotion, it does not reduce the psychological experience of that emotion, and may even intensify physiological responses by inhibiting expression, which in turn may be associated with stronger negative emotions and greater emotional difficulties (Lynch et al., 2001; Gross and John, 2003). This pattern was reflected in the present data. Whether driven by self-identity or group integration motivation, individuals who primarily relied on expressive suppression to align with group atmosphere or to conceal their true feelings may have continually drawn on their cognitive resources. Such sustained suppression may not only be associated with difficulty in genuinely internalizing emotion regulation skills, but may also be linked to a weaker sense of confidence in independently managing negative emotions when group support is unavailable (Lynch et al., 2001). When students become accustomed to suppressing their true feelings in order to maintain group integration, their regulatory emotional self-efficacy may conversely be lower.

In conclusion, the present study deepens theoretical understanding of how motivation relates to regulatory emotional self-efficacy and offers implications for mental health interventions among college students. However, this study also has several limitations. First, the present study is cross-sectional; future research could adopt longitudinal designs and integrate additional relevant factors to enhance the breadth and precision of the findings. Second, the use of convenience sampling limits representativeness; future studies could apply probability sampling methods, such as stratified random sampling, to test the robustness of these findings. Third, the 265 participants used to develop and validate the College Students’ “Wan Geng” Motivation Scale were a subset of the 1,084 participants used for hypothesis testing. This overlap may have introduced some optimistic bias (i.e., capitalization on chance) into the measurement and structural results, and future research should cross-validate the scale and the proposed model in an independent sample. Addressing these limitations would provide stronger support for understanding and improving college students’ emotion regulation.

## 5 Conclusion

Three findings emerged: (1) entertainment and group integration motivations were positively associated with emotion regulation self-efficacy; (2) “Wan Geng” motivations were linked to emotion regulation strategies; (3) entertainment, self-identity, and group integration motivations related to emotion regulation self-efficacy indirectly through emotion regulation strategies, while emotion regulation and entertainment motivations showed direct associations. These findings suggest that “Wan Geng” may play a constructive role in university students’ emotion regulation, deepening theoretical understanding of how motivation shapes regulatory emotional self-efficacy and offering implications for mental health interventions. An effective intervention may help students strengthen emotion regulation skills and transform positive experiences into psychological capital that supports their regulatory emotional self-efficacy.

## Data Availability

The datasets presented in this study can be found in online repositories. The names of the repository/repositories and accession number(s) can be found in the article/Supplementary material.
