# Supplementary material for: The influence of college students’ “Wan Geng” motivation on emotion regulation self-efficacy
Source: Front Psychol. 2026 Jul 17;17:1802652. doi: 10.3389/fpsyg.2026.1802652 (PMC13448810; doi:10.3389/fpsyg.2026.1802652)
Supplement: Supplementary file 1 [file Supplementary_file_1.pdf]

Supplementary Material

1 Development and Validation of the College Students' "Wan Geng" Motivation Scale

1.1 Item Development and Validation

Based on the four dimensions of college students' "Wan Geng" motivation (emotion regulation, entertainment, self-identity, and group integration), and drawing on relevant domestic and international research—including the online reading survey developed by Mondi et al. (2008) and the questionnaire on motivations underlying We Chat users' "liking" behavior developed by Rong et al. (2015), an initial item pool of 25 items was generated, with 4–5 items designed for each dimension. The initial questionnaire was then administered to selected respondents, and based on the survey results and feedback, the wording of certain items and the response options were further refined to produce the initial version of the College Students' "Wan Geng" Motivation Scale. The scale uses a 5-point Likert format (1 = strongly disagree, 5 = strongly agree), with higher scores indicating stronger "Wan Geng" motivation in the corresponding dimension. A small-scale pretest was conducted in March 2025, in which the online questionnaire was distributed to college students. After completeness checks and outlier screening, invalid responses were removed, yielding a final sample of 265 valid responses.

1.2 Exploratory Factor Analysis (EFA)

Since the College Students' "Wan Geng" Motivation Scale used in the present study was self-developed, it was necessary to test the reliability and validity of the scale following basic psychometric principles, in order to assess the appropriateness, reliability, and stability of each indicator as well as the overall scale in measuring the research constructs. Item analysis was conducted on the pretest data by calculating the critical ratio and the item-total correlation coefficient (r); items with a critical ratio < 3.00 or r < 0.30 were deleted. Exploratory factor analysis (EFA) was then performed to examine the structural validity of the scale. The KMO and Bartlett's tests were conducted on the pretest data, yielding a KMO value of 0.949 and a significant Bartlett's test of sphericity ($\chi^2 = 1023.56$, $p < 0.001$), which indicated adequate correlations among the constructs and the presence of common factors, suggesting that the data were suitable for factor analysis. Principal component analysis was used for factor extraction, with varimax rotation applied to identify the loadings of each item on the common factors using orthogonal rotation with Kaiser normalization.

Based on the EFA results, items were screened according to the criteria of factor loading > 0.50 and cross-loading < 0.35; items with loadings exceeding 0.40 on non-target dimensions were removed in sequence. Items 21, 20, 11, and 12 were first deleted. Because the remaining items were unevenly distributed across dimensions, correlation analysis was further conducted, and two additional items showing higher correlations with non-target dimensions were removed. Subsequently, items 5, 9, 10, 23, and 17 were deleted in turn based on the EFA results. After several rounds of testing and revision, the scale structure became stable, and the final version retained 14 items across four dimensions: emotion regulation motivation (3 items), entertainment motivation (3 items), self-identity motivation (4 items), and group integration motivation (4 items). All retained items had factor loadings above the recommended threshold of 0.50 (Orcan, 2018; Blaikie, 2003), which is within the acceptable

range, as presented in Supplementary Table 1. The total scale yielded a Cronbach's α of 0.945, and the α coefficients of all dimensions exceeded 0.70, indicating good internal consistency reliability.

1.3 Confirmatory Factor Analysis (CFA)

Confirmatory factor analysis (CFA) was conducted using Mplus 8.3 on the formal survey data to examine the structural validity of the self-developed College Students' "Wan Geng" Motivation Scale and to test the goodness-of-fit of the four-factor model with 14 items (N = 1,084). The CFA path diagram is presented in Supplementary Figure 1. The results indicated that all model fit indices reached satisfactory levels (Supplementary Table 2): CFI = 0.948 and TLI = 0.933, both exceeding the threshold of 0.90, suggesting that the scale demonstrated good model fit and that its structural validity was supported. The standardized factor loadings, composite reliability (CR), and average variance extracted (AVE) are reported in Supplementary Table 3. All standardized loadings exceeded 0.50, all CR values exceeded 0.70, and all AVE values exceeded 0.50, indicating adequate convergent validity and internal consistency reliability. Discriminant validity was assessed using both the Fornell–Larcker criterion and the heterotrait-monotrait (HTMT) ratio (Supplementary Table 4). The Fornell–Larcker criterion was satisfied for all dimension pairs except self-identity and group integration, for which the inter-factor correlation (.775) marginally exceeded the square roots of their AVEs (.758 and .710); however, the HTMT ratio for this pair (.746), and for all pairs (range = .493–.747), fell below the .85 threshold, supporting discriminant validity.

1.4 Reliability and Validity Analysis

The reliability and validity of the 14 self-developed items were examined using SPSS 30.0. To further verify the stability of the revised scale, 110 college students were randomly selected and retested two weeks later, as shown in Supplementary Table 5. The test-retest reliability of the College Students' "Wan Geng" Motivation Scale was 0.927, and the test-retest reliabilities for the four dimensions were 0.862, 0.857, 0.889, and 0.814, respectively, indicating good test-retest reliability. All correlations were statistically significant ($p < 0.01$). Taken together, the revised College Students' "Wan Geng" Motivation Scale demonstrated good reliability and validity, and can be used for large-scale assessment.

Supplementary Table 1. Exploratory Factor Analysis of the College Students' "Wan Geng" Behavior Motivation Scale.

Item \ Factor	Self-Identity Motivation	Emotion Regulation Motivation	Group Integration Motivation	Entertainment Motivation
No.1		**0.839**		
No.2		**0.791**		
No.4		**0.765**		
No.6				**0.695**
No.7				**0.796**
No.8				**0.806**
No.13	**0.771**			
No.14	**0.794**			
No.15	**0.683**			

Item \ Factor	Self-Identity Motivation	Emotion Regulation Motivation	Group Integration Motivation	Entertainment Motivation
No.16	**0.749**			
No.18			**0.662**	
No.22			**0.572**	
No.24			**0.781**	
No.25			**0.836**	

Note. N = 265. Extraction method: principal component analysis; rotation method: Kaiser-normalized varimax rotation. Item numbers correspond to the original 25-item pool; retained items were renumbered 1–14 in the final version of the scale.

Supplementary Table 2. Model Fit Indices of the Four-Factor CFA

χ^2	*df*	χ^2/df	CFI	TLI	RMSEA	SRMR
464.46	71	6.54	0.948	0.933	0.072	0.044

Note. CFI = comparative fit index; TLI = Tucker–Lewis index; RMSEA = root mean square error of approximation; SRMR = standardized root mean square residual.

Supplementary Table 3. Standardized Factor Loadings, Convergent Validity, and Reliability

Dimension	Item	Standardized loading	Cronbach's α	CR	AVE
**Emotion regulation**	1	.757	.838	.839	.635
	2	.840			
	3	.791			
**Entertainment**	4	.814	.812	.810	.588
	5	.717			
	6	.767			
**Self-identity**	7	.698	.844	.844	.575
	8	.729			
	9	.810			
	10	.791			
**Group integration**	11	.739	.800	.800	.504
	12	.801			
	13	.736			
	14	.536			

Note. N = 1,084. All standardized loadings were significant at $p < .001$. CR = composite reliability; AVE = average variance extracted. All loadings exceeded .50, all CR exceeded .70, and all AVE exceeded .50, indicating adequate convergent validity and reliability.

Supplementary Table 4. Discriminant Validity (Fornell–Larcker Criterion)

**Dimension**	**1**	**2**	**3**	**4**
**1. Emotion regulation**	**.797**			
**2. Entertainment**	.763	**.767**		
**3. Self-identity**	.602	.638	**.758**	
**4. Group integration**	.521	.564	.775	**.710**

Note. N = 1,084. Diagonal values (in bold) are the square root of the AVE for each dimension; off-diagonal values are inter-factor correlations from the CFA.

Supplementary Table 5. Test-Retest Reliability of the Revised College Students' "Wan Geng" Motivation Scale

	**Total Score**	**Factor 1 Emotion Regulation**	**Factor 2 Entertainment**	**Factor 3 Self-Identity**	**Factor 4 Group Integration**
Cronbach's α	0.961	0.931	0.841	0.906	0.889
Test-Retest Reliability	0.927**	0.862**	0.857**	0.889**	0.814**

Note. N = 110. **Correlation is significant at the 0.01 level (2-tailed). Correlations are Pearson correlation coefficients.

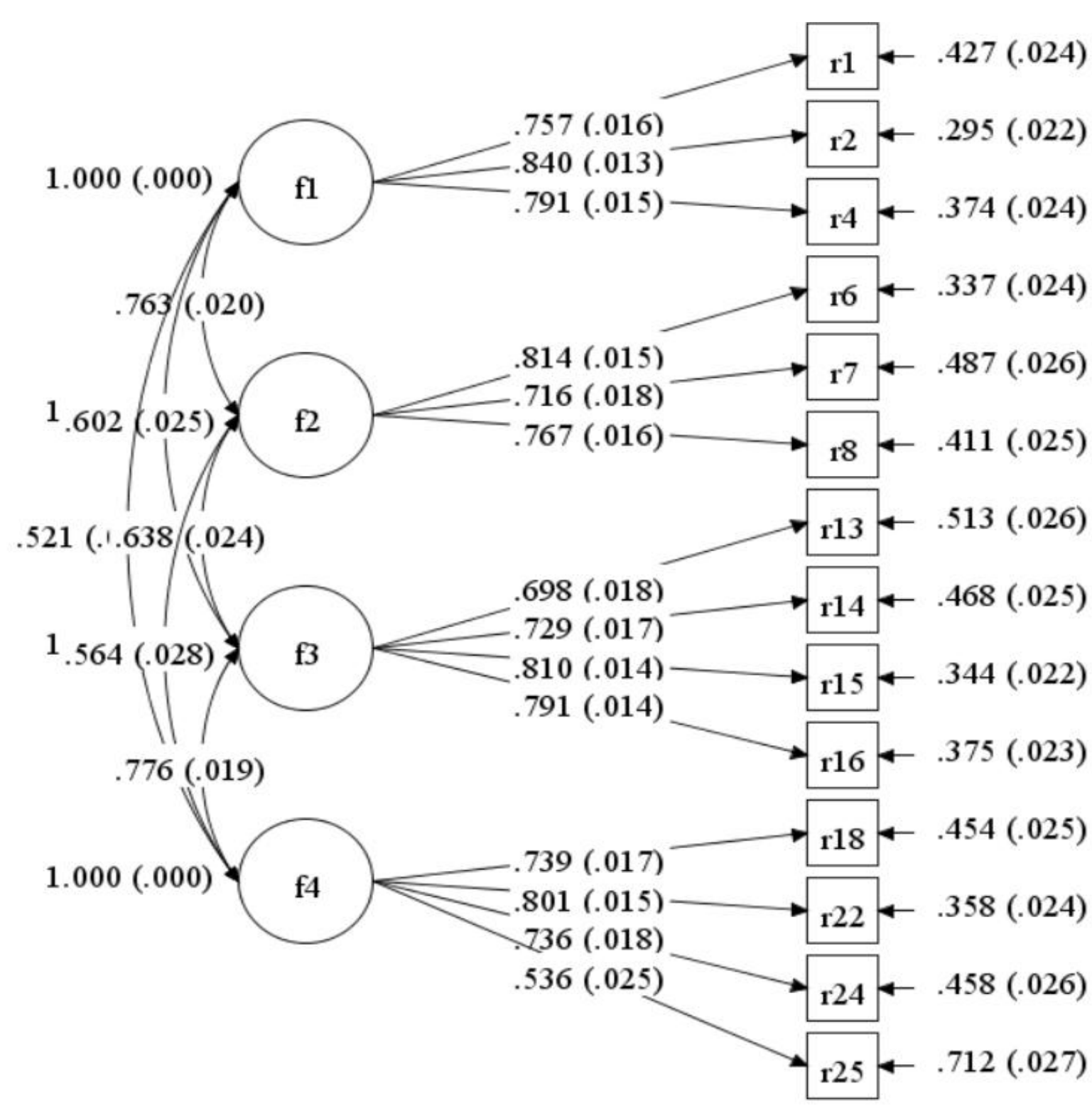

Supplementary Figure 1. CFA of the "Wan Geng" Motivation Scale

2 Motivation Scale for College Students' "Wan Geng" Behavior (Self-compiled)

Instructions:

Please check "√" the option that best corresponds to your actual situation. (Note: All items are single-choice questions. 1=Strongly Disagree, 2=Disagree, 3=Uncertain, 4=Agree, 5=Strongly Agree)

No.	Item	SD	D	U	A	SA
1	"Wan Geng" allows me to vent my frustration.	1	2	3	4	5
2	"Wan Geng" relieves my stress or tension.	1	2	3	4	5
3	"Wan Geng" enables me to temporarily escape the troubles of real life and release pressure.	1	2	3	4	5
4	"Wan Geng" brings me joy.	1	2	3	4	5
5	"Wan Geng" satisfies my interests and hobbies.	1	2	3	4	5
6	I reference "Geng" because it is interesting and appealing.	1	2	3	4	5
7	"Wan Geng" allows me to express my stance and attitude.	1	2	3	4	5
8	"Wan Geng" can directly reflect my current state.	1	2	3	4	5
9	"Wan Geng" makes me more humorous, enhances my personal charm, and makes me more confident.	1	2	3	4	5
10	"Wan Geng" helps to shape and enhance my personal image.	1	2	3	4	5
11	People around me reference "Geng", which facilitates understanding and communication.	1	2	3	4	5
12	"Wan Geng" allows me to better maintain interpersonal relationships and enhance social interaction.	1	2	3	4	5
13	"Wan Geng" makes me seem sociable (or helps me fit into a certain group).	1	2	3	4	5
14	Sometimes, when everyone else is referencing a "Geng", I feel compelled to understand and reference it too.	1	2	3	4	5
